# Voluntary and Involuntary Attention in Bistable Visual Perception: A MEG Study

**DOI:** 10.3389/fnhum.2020.597895

**Published:** 2020-12-22

**Authors:** Parth Chholak, Vladimir A. Maksimenko, Alexander E. Hramov, Alexander N. Pisarchik

**Affiliations:** ^1^Center for Biomedical Technology, Technical University of Madrid, Pozuelo de Alarcón, Madrid, Spain; ^2^Laboratory of Neuroscience and Cognitive Technology, Center for Technologies in Robotics and Mechatronics Component, Innolpolis University, Innopolis, Russia; ^3^Department of Automation, Control and Mechatronics, Saratov State Medical University, Saratov, Russia

**Keywords:** magnetoencephalography (MEG), attention, visual perception, brain noise, wavelet analysis (WA)

## Abstract

In this study, voluntary and involuntary visual attention focused on different interpretations of a bistable image, were investigated using magnetoencephalography (MEG). A Necker cube with sinusoidally modulated pixels' intensity in the front and rear faces with frequencies 6.67 Hz (60/9) and 8.57 Hz (60/7), respectively, was presented to 12 healthy volunteers, who interpreted the cube as either left- or right-oriented. The tags of these frequencies and their second harmonics were identified in the average Fourier spectra of the MEG data recorded from the visual cortex. In the first part of the experiment, the subjects were asked to voluntarily control their attention by interpreting the cube orientation as either being on the left or right. Accordingly, we observed the dominance of the corresponding spectral component, and voluntary attention performance was measured. In the second part of the experiment, the subjects were asked to focus their gaze on a red marker at the center of the cube image without putting forth effort in its interpretation. The alternation of the dominant spectral energies at the second harmonics of the stimulation frequencies was treated as changes in the cube orientation. Based on the results of the first experimental stage and using a wavelet analysis, we developed a method which allowed us to identify the currently perceived cube orientation. Finally, we characterized involuntary attention using the distribution of dominance times when focusing attention on one of the cube orientations, which was related to voluntary attention performance and brain noise. In particular, we confirmed our hypothesis that higher attention performance is associated with stronger brain noise.

## 1. Introduction

Wilhelm Wundt was the first to suggest, in as early as in 1897, that two forms of attention exist: voluntary and involuntary (Wundt, 1897). There is already more than a justifiable number of terms used in the community that overlap with these two forms of attention, such as endogenous vs. exogenous attention, automatic vs. controlled attention, and pull vs. push attention (Prinzmetal et al., 2005). According to Prinzmetal and his colleagues, voluntary and involuntary attention have different functions and are controlled by distinct mechanisms (Prinzmetal et al., 2005). They supposed that voluntary attention affects perceptual attention and would affect both accuracy and reaction time (RT) experiments, whereas involuntary attention deals with the response-selection decision and is manifested only in RT experiments. To study these differences, Posner et al. (1978), Posner (1978), Posner et al. (1980), and Posner (1980) developed a spatial cuing task. In their method, the subjects were asked to detect or identify a peripheral stimulus. The participants were pre-cued to a possible location of the stimulus beforehand; in valid trials, the cue indicated the target location, whereas in the case of invalid trials, the cue indicated a non-target location. Since the participants were not allowed to move their eyes to the cued location, the observed differences in performance between valid and invalid trials reflected differences in attention which were independent of fixation. Later, Jonides (1980) and Jonides (1983) used this paradigm to study the difference between voluntary and involuntary attention by altering the “validity” of the cuing information. If the total number of valid trials for the correct stimulus location is as low as that for a random distribution in which no useful bias for the target location is provided, only involuntary attention would be involved in seeing the peripheral stimulus. On the other hand, in the presence of a high number of valid trials in which correct cuing information for the target location is available, both voluntary and involuntary attention would be engaged.

In 2005, Prinzmetal et al. (2005) introduced the idea of channel enhancement and channel selection in order to show how the two kinds of attention manifest. Channel enhancement is a process driven by voluntary attention that causes the visual system to gather more information from the attended stimulus than from the unattended stimulus specified by the informative cues. It changes the perceptual representation so that the observers have a clearer view of the stimulus they are attending to Prinzmetal et al. (1997a), Prinzmetal et al. (1997b), and Prinzmetal et al. (1998). Other researchers also confirmed that attention to the biasing cue improves the perceived contrast of both attended and unattended stimuli (Carrasco et al., 2004; Luck, 2004; Treue, 2004).

There is a general consensus that the Stroop effect alters the response selection, but not perceptual representation (Virzi and Egeth, 1985; Baldo et al., 1998). For example, when shown the word BLUE written in red and asked the color, it would lead to a competition in the response selection that delays the response, but no alteration in the perceived color would be observed. Similarly, involuntary attention would affect RT, but not detection accuracy. Conveniently, several researchers reported that involuntary attention to a stimulus only affects the response selection (Ooi and He, 1999; Mitchell et al., 2004; Hancock and Andrews, 2007).

It should be noted that there is a precedence for accuracy and RT studies to produce opposing effects (Santee and Egeth, 1982; Mordkoff and Egeth, 1993; Moore and Egeth, 1998). In particular, Santee and Egeth (1982) considered the redundant target paradigm, in which a target letter is repeated on a display. They found that the repeating target speeds up the reaction (Eriksen and Eriksen, 1974, 1979; Eriksen and Schultz, 1979) but reduces the accuracy (Bjork and Murray, 1977; Santee and Egeth, 1980). This phenomenon is known as the flanker effect. The correct selection of recording channels should also alter the detection accuracy in the target location which is being attended. Furthermore, it may also improve RT as information is presumably gathered faster in the cued than in the uncued location. Moreover, channel selection deals with decision making when determining the correct target location or response selection, and only affects RT experiments.

In this paper, we study voluntary and involuntary attention using multistable perception (Leopold and Logothetis, 1999), a phenomenon where the same stimulus can be perceived in more than one way. With regard to degrees of freedom, the simplest form of multistable perception is bistable perception: when two different interpretations of the same stimulus are possible. As a result of extensive research on this topic over the last two decades, many descriptive models were developed (Moreno et al., 2007; Shpiro et al., 2009; Huguet et al., 2014; Dotov et al., 2019; Meilikhov and Farzetdinova, 2019; Chholak et al., 2020a). The switches between alternate percepts were suggested to be driven by either stochastic processes in the brain (Moreno et al., 2007; Pisarchik et al., 2014) due to random neurophysiological activity and neuronal adaptation (Huguet et al., 2014; Dotov et al., 2019), which is defined as slow destabilization of currently dominant perception after being active for a prolonged time, or due to both noise and adaptation (Shpiro et al., 2009; Huguet et al., 2014; Chholak et al., 2020a). Each percept competes with another rival state, while the dominant active state tends to suppress alternative perception. Several researchers also studied visual attention modulation in the striate and extrastriate visual cortex (Hillyard and Anllo-Vento, 1998; Mangun et al., 1998; Brefczynski and De Yoe, 1999; Ghandi et al., 1999; McAdams and Maunsell, 1999; Reynolds and Desimone, 1999, 2003; Reynolds et al., 2000; Treue, 2000; Martinez-Trujillo and Treue, 2002; Saenz et al., 2002). Whether the interstate suppression comes before binocular confluence, such as in the primary visual cortex or the lateral geniculate nucleus (Blake, 1989; Lehky and Blake, 1991; Tong and Engel, 2001), or after binocular confluence (Logothetis et al., 1996; Andrews and Purves, 1997) was a matter of numerous debates. The latter assumes that there is a competition between high-level stimulus representations in visual neurons (Ooi and He, 1999; Hancock and Andrews, 2007).

Similarly, the phenomenon of visual attention is based on the competition of one object among a variety of other competing alternatives for enhanced perceptual representation as in voluntary attention. This leads to the suggestion that bistable perception and attention may be related processes (Helmholtz, 1962; Leopold and Logothetis, 1999). Previous studies on this topic were performed using evoked responses that consisted of numerous relatively short trials as opposed to a single long trial. The present work, on the contrary, is aimed to characterize voluntary and involuntary attention using visual responses from relatively long (120-s) trials. Furthermore, involuntary attention was only found in RT experiments under the evoked response regime. Instead of the evoked response, we use long entrained visual signals that can vary in phase and hence are unlocked in time with the start of stimulation. The corresponding brain response is termed as *visual induced field* (VIF), in contrast to the traditionally used visual evoked field (VEF). In the first part of our experiment we study controlled (voluntary) attention, when subjects are asked to fix their attention on one of two possible Necker cube orientations, whereas in the second part we investigate involuntary attention when subjects do not try to interpret (control) the cube orientation. We measure the subject's attentional ability in the first part and use the gained insight in the second part to estimate involuntary attention based on the method of wavelet energies. Finally, we characterize involuntary attention using dominance time distribution and study its relation to voluntary attention performance and brain noise.

## 2. Materials and Methods

### 2.1. Experimental Setup

Magnetoencephalographic (MEG) data were recorded with a whole-head Vectorview MEG system (Elekta AB, Stockholm, Sweden) with 306 channels (102 magnetometers and 204 planar gradiometers), which were placed inside a magnetically shielded room (Vacuum Schmelze GmbH, Hanau, Germany) at the Laboratory of Cognitive and Computational Neuroscience, Center for Biomedical Technology, Technical University of Madrid, Spain. Fastrak digitizers (Polhemus, Colchester, Vermont) were used to obtain a three-dimensional head shape using approximately 300 points on the scalp of each subject. Additionally, three fiducial points (nasion, left and right pre-auricular) were acquired for co-registration purposes. A vertical electrooculogram was placed to capture eye blinks. A single empty room recording lasting more than 2 min was performed on each day of the experiment (Day-1: 4 subjects; Day-2: 5 subjects; Day-3: 3 subjects). Data were sampled at 1,000 Hz with an on-line anti-alias bandpass filter between 0.1 and 330 Hz.

### 2.2. Participants

Twelve^1^ healthy subjects (aged 17–64 years, six males and six females) with normal or corrected-to-normal vision participated in the experimental study. All subjects provided written informed consent before the commencement of the experiment. The experimental studies were performed in accordance with the Declaration of Helsinki and approved by the Ethics Committee of the Technical University of Madrid.

### 2.3. Visual Stimulus

**Figure 1:**
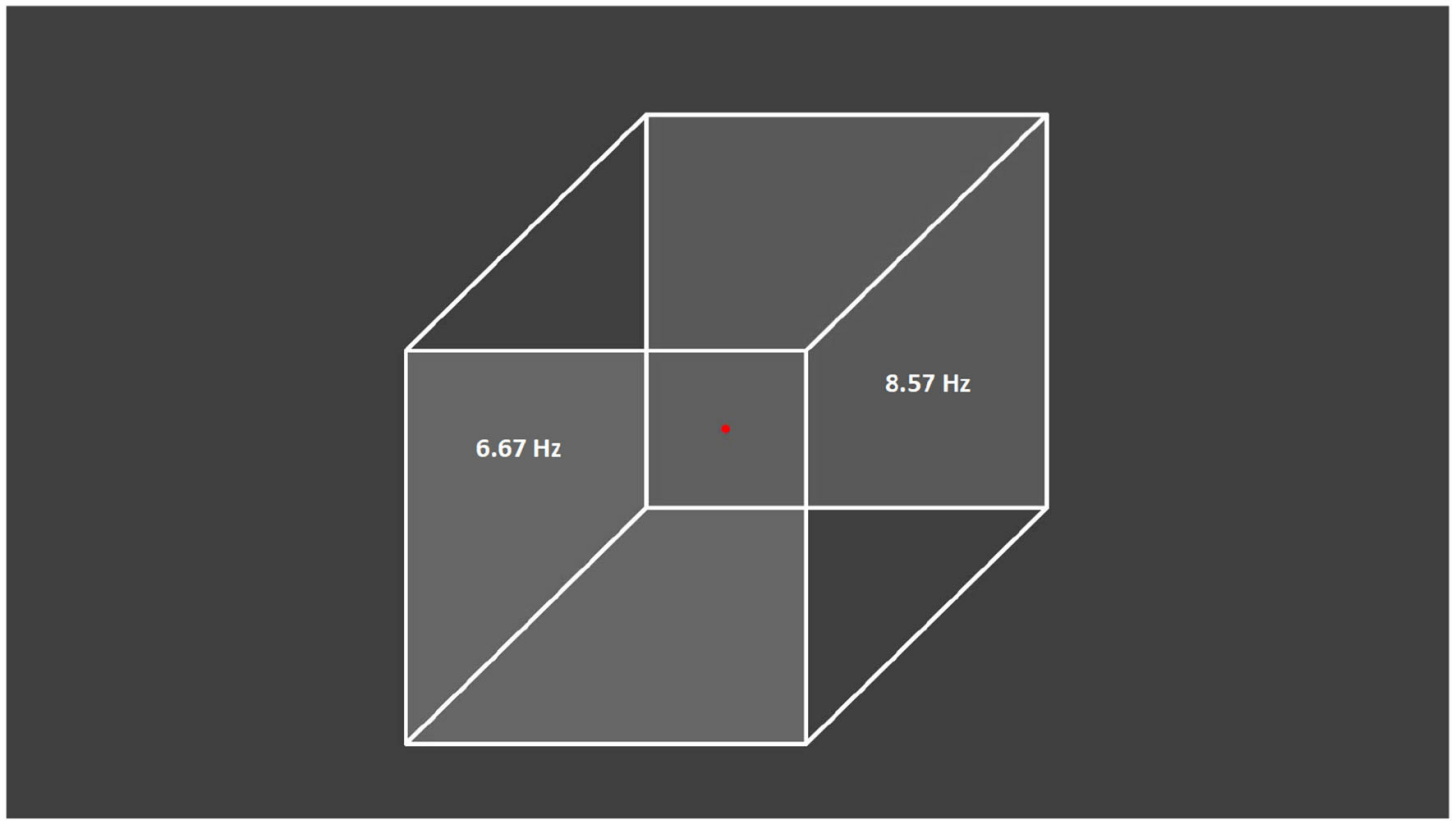
Presented visual stimulus. Necker cube with flickering left and right faces at 6.67 and 8.57 Hz, respectively. The subjects were asked to fix their gaze on the central red dot.

The visual stimulus was a gray Necker cube image on a gray background drawn on a computer monitor with a 60-Hz refresh rate and subsequently projected by a digital light processing projector onto a translucent screen located 150 cm away from the subject (Figure 1). The pixels' brightness on the left- and right-cube front faces was modulated by sinusoidal signals with 6.67-Hz (60/9) and 8.57-Hz (60/7) frequencies, respectively. The modulation depth was 100% with respect to the medium gray-scale level of the pixels' brightness (127 in an 8-bit format), i.e., the image brightness varied from black (0) to gray (127). The sinusoidal shape and modulation frequencies were chosen in preliminary experiments where different signal shapes (sinusoidal, rectangular and triangular) and different flicker frequencies which are integral fractions of the 60-Hz frame rate (i.e., 60/2, 60/3, 60/4, 60/5, 60/6, 60/7, 60/8, 60/9, 60/10, and 60/12) were explored. The selected frequencies produced the best tags in the brain response (Pisarchik et al., 2019a).

To perform the experiment, including the presentation of the visual stimulus, we designed a special algorithm using the Cogent Graphics MATLAB Toolbox publicly available on GitHub®. Data acquisition was made by the software provided with the Elekta-Neuromag system, while the time-stamps corresponding to the onset of visual stimulus presentation were marked on-line using a parallel port. The MATLAB code also included these event markers via parallel port connection. For more details, see the section on code and data sharing.

### 2.4. Experimental Procedure

The subjects were seated in a comfortable reclining chair with their legs straight and arms resting on an armrest in front or on their laps. The participants were asked to remove any metallic items above their waist like jewelry, belts, and brassieres, along with their shoes prior to the experiment. The experiment began with the recording of a 2-min background activity while the subject was focusing on a red dot located in the middle of a stationary (non-flickering) cube image. This MEG trial acted as a background reference in further analyses.

The entire experiment included two stages: voluntary control of the perceived cube orientation and involuntary spontaneous switching between two cube orientations. During the first stage, after a 30-s rest and an instructional visual message, a flickering Necker cube with two frequencies was presented 24 times on the screen (5 s each with a 5-s interval gap in-between). For the first 12 trials, 9 out of 12 participants were asked to interpret the cube as left-oriented. After a 30-s rest and an instructional visual message, the participants were requested to interpret the next 12 cubes as right-oriented. For 3 subjects, we reversed the order of voluntary perception by asking them to interpret the first 12 cubes as right-oriented and the next 12 cubes as left-oriented. This concluded the first experimental stage.

The second part of the experiment started with the same Necker cube presentation but now for 120 s. At this stage, the subjects were instructed not to fix their attention on any particular cube orientation. In all of the experiments, the participants were asked to focus only on the red dot at the center of the image. This was done to ensure that changes in the cube orientation were not caused by eye movements, i.e., at the retinal level, but instead by visual neurons at a higher level.

### 2.5. Visual Induced Field (VIF)

In this paper, we introduce a new measure of visual brain response which is defined as the average brain activity in the visual cortex for longer than transient-evoked-response time durations that are not phase-locked to the stimulus. The traditionally used visual evoked field (VEF) is time-locked to the stimulus and thus one averages visual brain activity across trials. Since we observe intermediate phase-slips in experiments with longer (e.g., 120-s) durations at unpredictable time moments, averaging across multiple trials is not possible. Although this response is caused by the stimuli, the time moments of its start and intermediate phase-slips are not fixed. Therefore, we aptly named it *visual induced response* (VIR) opposed to visual evoked response (VER). It should be noted that VER at such time scales, averaged over multiple trials, is called steady-state visual evoked response (SSVER).

The brain was mapped using a mesh of 15,004 points representing cortical sources. There are multiple combinations in which these numerous brain sources can produce the observed magnetic activity recorded by 306 MEG channels. This so-called inverse problem is ill-posed and can only be solved by using additional assumptions about the neuronal system such as minimization of the total energy of the system. The depth-dependent sensitivity and spatial resolution were normalized using the standardized low-resolution electromagnetic tomography (sLORETA) method.

After aligning a standard anatomical magnetic resonance imaging (MRI) provided in Brainstorm (Tadel et al., 2011) with the fiducial points, the cranial anatomy was warped to fit the acquired points on the scalp using the Polhemus device with an error margin of 2%. We used the Brodmann atlas in Brainstorm to find cortical sources associated with visual areas V1 and V2 on the modeled cortical mesh (1,227 points). The response of these visual sources was then averaged to obtain VIF for each trial.

### 2.6. Spectral Analysis

Morlet-based wavelets constructed from a mother wavelet with a 1-Hz central frequency and a 12-s full width at half maximum (FWHM) were utilized to obtain wavelet power time series at the second harmonics of the flicker frequencies. The second harmonic frequencies were fine-tuned based on the power spectrum of the VIF signals for each subject.

### 2.7. Wavelet Analysis

The time-frequency analysis is based on the continuous wavelet transform (Pavlov et al., 2012; Hramov et al., 2015)

(1) $$\begin{array}{l} W\left(f,t\right)=\sqrt{f}\int_{t-4/f}^{t+4/f}X\left(t\right)\psi^{*}\left(f,t\right)dt, \end{array}$$

where “*” denotes the complex conjugate and *X*(*t*) is the analyzed MEG signal. The complex-valued Morlet-wavelet is chosen as the mother wavelet

(2) $$\begin{array}{l} \psi \left(f,t\right)=\sqrt{f}\pi^{1/4}e^{i\omega_{0}f\left(t-t_{0}\right)}e^{f{\left(t-t_{0}\right)}^{2}/2}, \end{array}$$

with ω_0_ = 2π*f*_0_ being the central frequency of the Morlet wavelets and $i=\sqrt{-1}$. The wavelet powers *W*(*f*_1_, *t*) and *W*(*f*_2_, *t*) given by Equation (1) were evaluated at the tagging frequencies *f*_1_ = 13.33 Hz and *f*_2_ = 17.14 Hz (second harmonics of the flicker frequencies), respectively. Since the frequency response decays with increasing frequency as a 1/*f* rule, the wavelet energy is normalized to the corresponding modulation period (1/*f*_1, 2_). Hence, the wavelet time series are multiplied to their defining frequencies to get

(3) $$\begin{array}{l} E_{1}=W\left(f_{1},t\right)f_{1}\text{ and }E_{2}=W\left(f_{2},t\right)f_{2} \end{array}$$

and the difference between the spectral energies at *f*_1_ and *f*_2_ is then calculated as

(4) $$\begin{array}{l} \Delta E=E_{1}-E_{2} \end{array}$$

and normalized to its maximum absolute value as

(5) $$\begin{array}{l} \bar{\Delta E}=\Delta E/\max|\Delta E|. \end{array}$$

**Figure 2:**
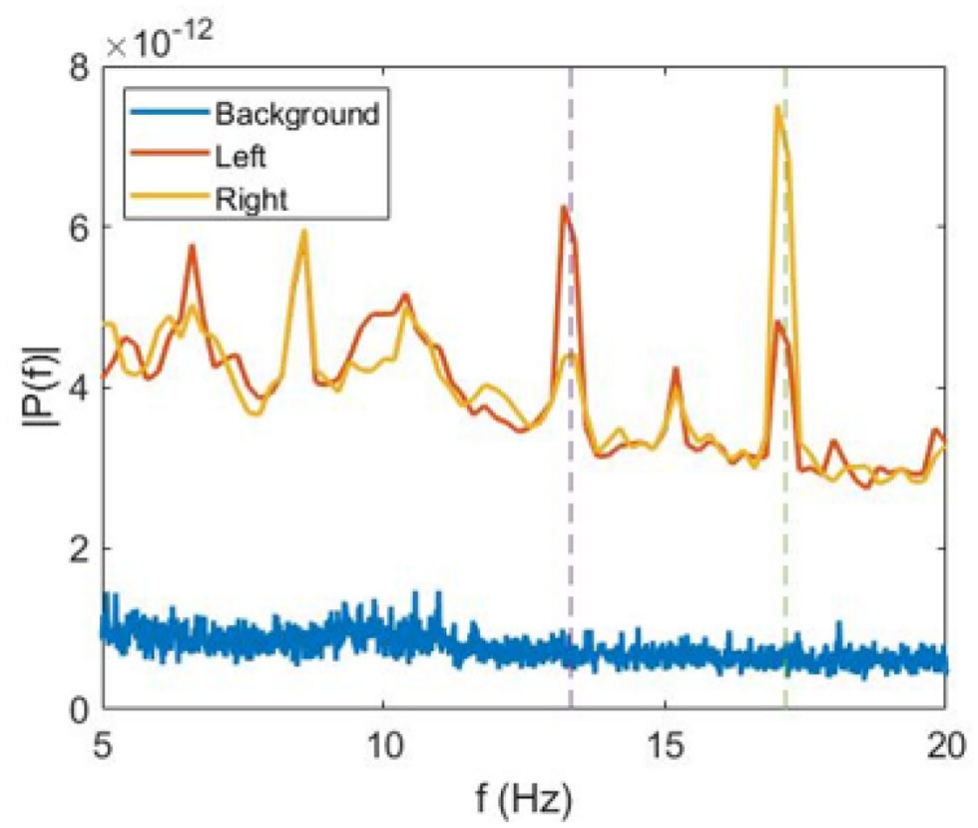
Average power spectra. Fourier spectra of VIF averaged over all subjects during perception of non-flickering (background) (blue), left-oriented (red), and right-oriented (green) cubes.

In our analysis, we averaged *E*_1_ and *E*_2_ over time and over all trials separately for the left-oriented ($P_{1}^{L}$ and $P_{2}^{L}$) and for the right-oriented ($P_{1}^{R}$ and $P_{2}^{R}$) cube interpretations. The power spectra averaged over all participants are shown in Figure 2.

**Figure 3:**
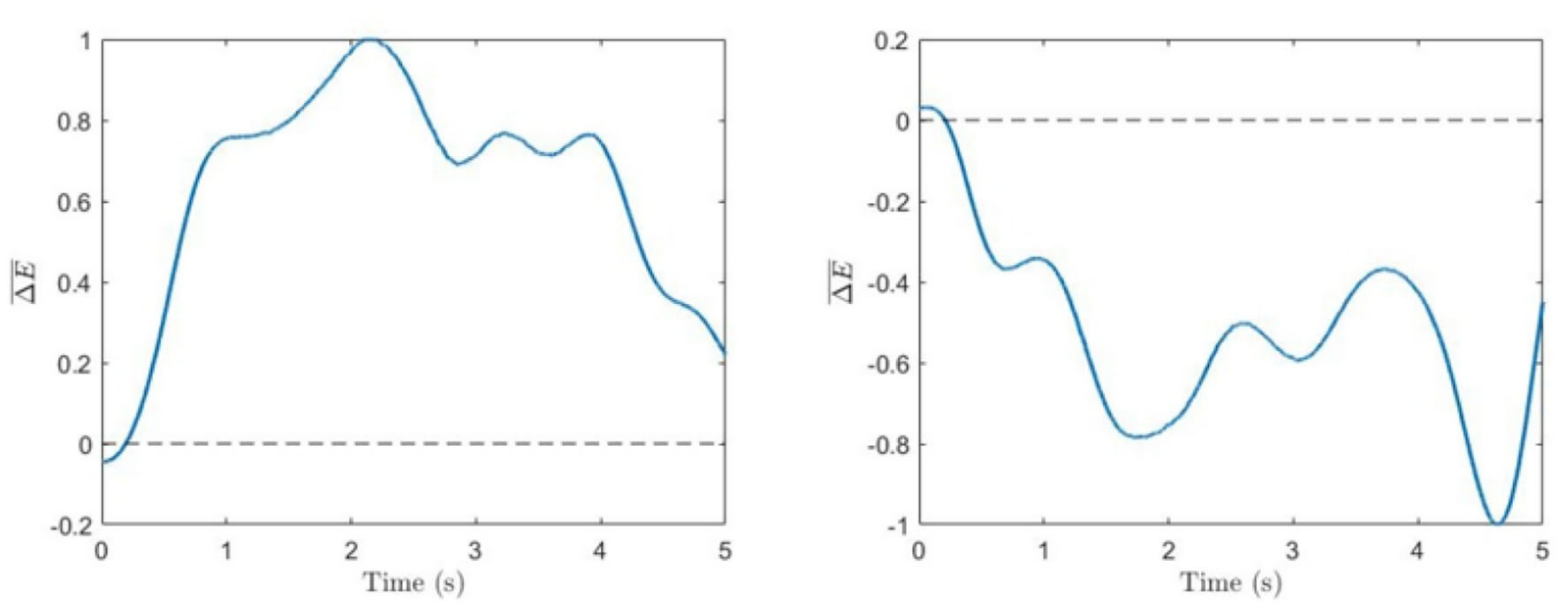
Spectral difference. Time series of normalized energy difference (Equation 5) for single trials corresponding to voluntary left-oriented **(left)** and right-oriented **(right)** cube perception.

The evolution of the normalized energy difference in Equation (5) for typical 5-s trials corresponding to the left and right cube orientations for one of the subjects is shown in Figure 3.

The differences between the wavelet energies at *f*_1_ and *f*_2_ corresponding to the left-oriented and right-oriented cube perceptions ($D_{1,2}=P_{1,2}^{L}-P_{1,2}^{R}$) signify the bias in spectral reflection of left orientation in comparison to right orientation such that *D*_1_ should be higher and *D*_2_ should be lower. The difference between *D*_1_ and *D*_2_ defines the performance index μ as

(6) $$\begin{array}{l} \mu =D_{1}-D_{2}. \end{array}$$

The performance μ characterizes the ability of the subject to voluntarily attend to the foretold cube orientation. Similar to the voluntary case, normalized energy difference time series for both frequencies were evaluated from VIF for involuntary perception. However, unlike the voluntary case, the trial duration was increased to 120 s.

### 2.8. Marking Perception States

To determine the moment of switching between two different cube orientations, we propose a method based on wavelet power time series. In our approach, $\bar{\Delta E}$ calculated by Equation (5) is screened for significant changes above a threshold equal to its standard deviation δ:

(7) $$\begin{array}{l} \bar{\left|\Delta E\right|}>\delta . \end{array}$$

**Figure 4:**
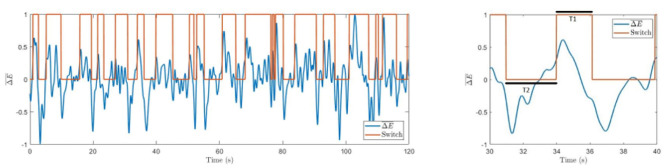
Difference in spectral energies. Time series with marked switches between involuntary left-oriented and right-oriented cube perception. The right panel shows an enlarged part of the left graph with marked resting times T1 and T2.

The active state is determined as left-oriented (Switch = 1) if $\bar{\Delta E}>\delta$ and as right-oriented (Switch = 0) if $\bar{\Delta E}<-\delta$. The algorithm is resilient to insignificant perturbations and sticks to the previous state for $-\delta <\bar{\Delta E}<\delta$. Typical switches in perception between the two cube orientations are illustrated in Figure 4.

### 2.9. Event-Related Coherence

In order to localize the brain sources during the second part of the experiment, we calculated event-related coherence (ERC).

To reduce the computational load, we first stripped the 120-s trials into forty 3-s trials for both the second part of the experiment and the background recordings. For each of the stripped trials for both experimental conditions, the magnitude-squared coherence between the estimated source time series (15,004 signals) and a sinusoidal signal at either of the tagging frequencies, i.e., *f*_1_ or *f*_2_, was evaluated. After averaging the coherence values over all forty trials, the difference between average coherence during the second part of the experiment and background was calculated and termed as ERC. The ERC was thus computed over all 15,004 brain sources to generate heat maps for source localization. For a detailed description of the method, see (Chholak et al., 2020b).

The ERC maps were evaluated at both tagging frequencies, *f*_1_ and *f*_2_, and then averaged to give the final source localization map.

## 3. Results and Discussion

### 3.1. Experiment-1: Voluntary Control of Perceived Cube Orientation

We observe that for the left-oriented cube interpretation the spectral energy is higher at *f*_1_ than at *f*_2_, whereas for the right-oriented cube the opposite situation occurs. This can be seen in Figure 2, where we plot the power spectra averaged over all subjects during trials with the left-oriented cube, right-oriented cube, and stationary cube (background) without flickering.

Hence, we expect *dominance* of the left orientation over the right orientation, calculated as the difference between the spectral powers corresponding to two different cube orientations, at *f*_1_ (or *D*_1_) to be positive and higher than at *f*_2_ (or *D*_2_), which should be negative and lower than *D*_1_. Furthermore, the difference between *D*_1_ and *D*_2_ would signify the performance in subject's voluntary attention (μ) to tend to perceive both cube orientations, because the reason for perceiving the contrast between the attended and unattended stimuli is voluntary attention.

Figure 3 shows typical times series of the spectral power difference for the left- and right-face frequencies during voluntary attention on the left- and right-cube orientations. In Table 1, we present the dominance of the left orientation over the right orientation for both frequencies and voluntary attention performance μ calculated by Equation (6). While the spectral difference *D*_1_ is marginally positive, *D*_2_ is largely negative. One can see that μ is always positive.

**Table 1:** Dominance of left-oriented (*D*_1_) over right-oriented (*D*_2_) cube interpretations at *f*_1_ and *f*_2_, respectively, and voluntary attention performance (μ).

**Subject**	***D*_1_**	***D*_2_**	**μ**
A	1.151	−1.138	2.289
B	1.620	−0.964	2.584
C	0.358	−1.027	1.385
D	0.857	−0.524	1.380
E	0.005	−0.612	0.618
F	0.250	−1.299	1.549
G	0.059	−0.287	0.346
H	−0.120	−0.548	0.427
I	−0.152	−0.998	0.846
K	0.530	−1.041	1.570
L	0.675	−0.992	1.667
Mean (σ)	0.476 (0.562)	−0.857 (0.313)	1.333 (0.724)

As discussed in Introduction, the influence of attention on contrast sensitivity is well-documented by various experiments. The remaining question is whether the enhancement in contrast is due to an increase in the dominance of an attended stimulus (Chong et al., 2005) or a decrease in the dominance of an unattended stimulus (Carrasco et al., 2004; Hancock and Andrews, 2007). Many studies claim that attention enhances perceptual sensitivity (Prinzmetal et al., 1997a, 1998; Lu and Dosher, 1998; Carrasco et al., 2000; Cameron et al., 2002). In this regard, two prominent models were proposed. One of them implies that attention improves the quality of neural response to the stimulus (signal enhancement) (Carrasco et al., 2000, 2002; Cameron et al., 2002), while the other suggests that attention reduces the response to an unattended stimulus (external noise reduction) (Lu and Dosher, 1998; Baldassi and Burr, 2000). In their pioneering work, Carrasco et al. (2004) demonstrated with a clever set of psychophysical experiments on a large number of subjects that attention enhances the strength of the perceived stimulus by reducing the impact of unattended stimuli.

Our findings also support the attentional mechanism of external noise reduction as opposed to signal enhancement. When the subject is voluntarily attending to the left-oriented cube in comparison to the right-oriented cube, the dominance of the attended stimulus frequency *f*_1_ does not increase as much as there is a decrease in the unattended stimulus frequency *f*_2_. Thus, the enhanced contrast of the attended to the unattended stimulus due to voluntary attention is caused by a decrease in the unattended stimulus dominance.

However, it is noteworthy that Carrasco et al. (2000), Cameron et al. (2002), and Carrasco et al. (2002) worked with the paradigm of transient attention which was infused using visual cues and lasted for up to a maximum of only 250 ms. In our study, the subjects were asked to maintain their attention during the entire period of 5-s trials. Therefore, the above mechanism is not only true for transient responses but also for sustained long-term responses.

### 3.2. Experiment-2: Involuntary Switches Between Different Perceptual States

**Figure 5:**
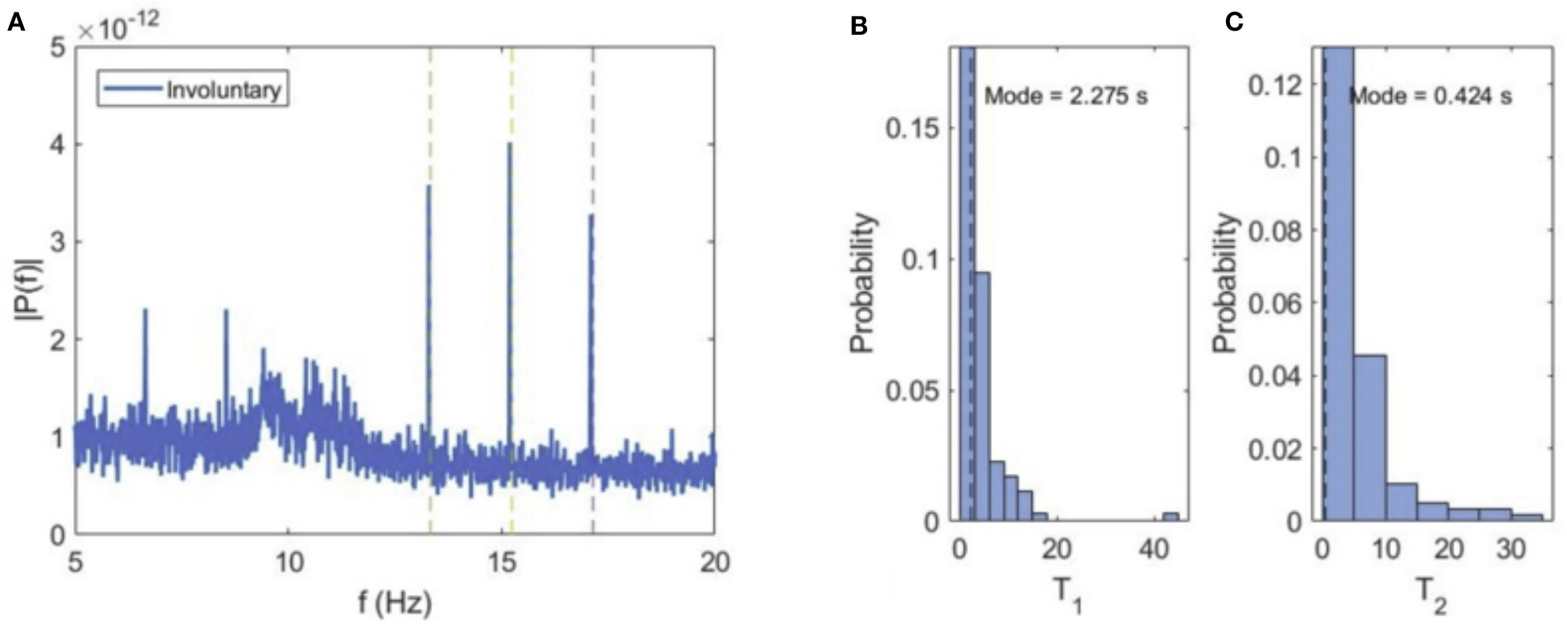
Involuntary attention. **(A)** Average power spectrum of VIF for all subjects during involuntary switching between the two cube orientations. **(B,C)** Probability distributions of dominance times for left (T_1_) and right (T_2_) cube orientations.

When the subjects spontaneously switch their attention to either of the cube orientations, the VIF spectral content exhibits narrow peaks at tagging frequencies *f*_1_ and *f*_2_ and sum flicker frequencies (*f*_1_ + *f*_2_)/2 (Figure 5). This can be explained by the fact that during perception of either of the cube orientations, the central square at the intersection of both orientations is flickering at the superposition frequency, and is consequently attended during the perception of either orientation.

The average values of dominance times for both orientations are similar (*T*_*a*1_ = 4.097 ms, *T*_*a*2_ = 5.124 ms), but curiously, the most probable or modal dominance time for the left orientation (*T*_*m*1_ = 2.275 s) is much higher than for the right orientation (*T*_*m*2_ = 0.424 s). This seems to suggest a bias in the perception of the two cube orientations, i.e., the same stimulation excites the left orientation more easily and frequently than the right orientation.

Perception selection can be affected by spatial, ocular, or feature-based mechanisms. In our study, both cube perceptions were shown to both eyes and so the interocular competition did not affect orientation selection. Since the subject's eyes were fixated to the central red dot from which both cube skeletons were symmetrically located, we can also rule out spatial selection. Lastly, the features of both orientations were identical and came out on the screen together abruptly without any smooth transitions between them. Hence, we can also rule out feature-based mechanisms.

Another possible reason for the preference of the left-cube orientation can be that in our everyday lives, we see the left-oriented cube more often and hence the perceptual stability of the left-cube orientation is higher (Chholak et al., 2020a). This form of attention in perceptual selection that does not depend upon ocular, spatial, or feature-based mechanisms but solely on the representational object it corresponds to, is called *object-based attention* and has shown to determine dominance in bistable perception (Mitchell et al., 2004).

**Figure 6:**
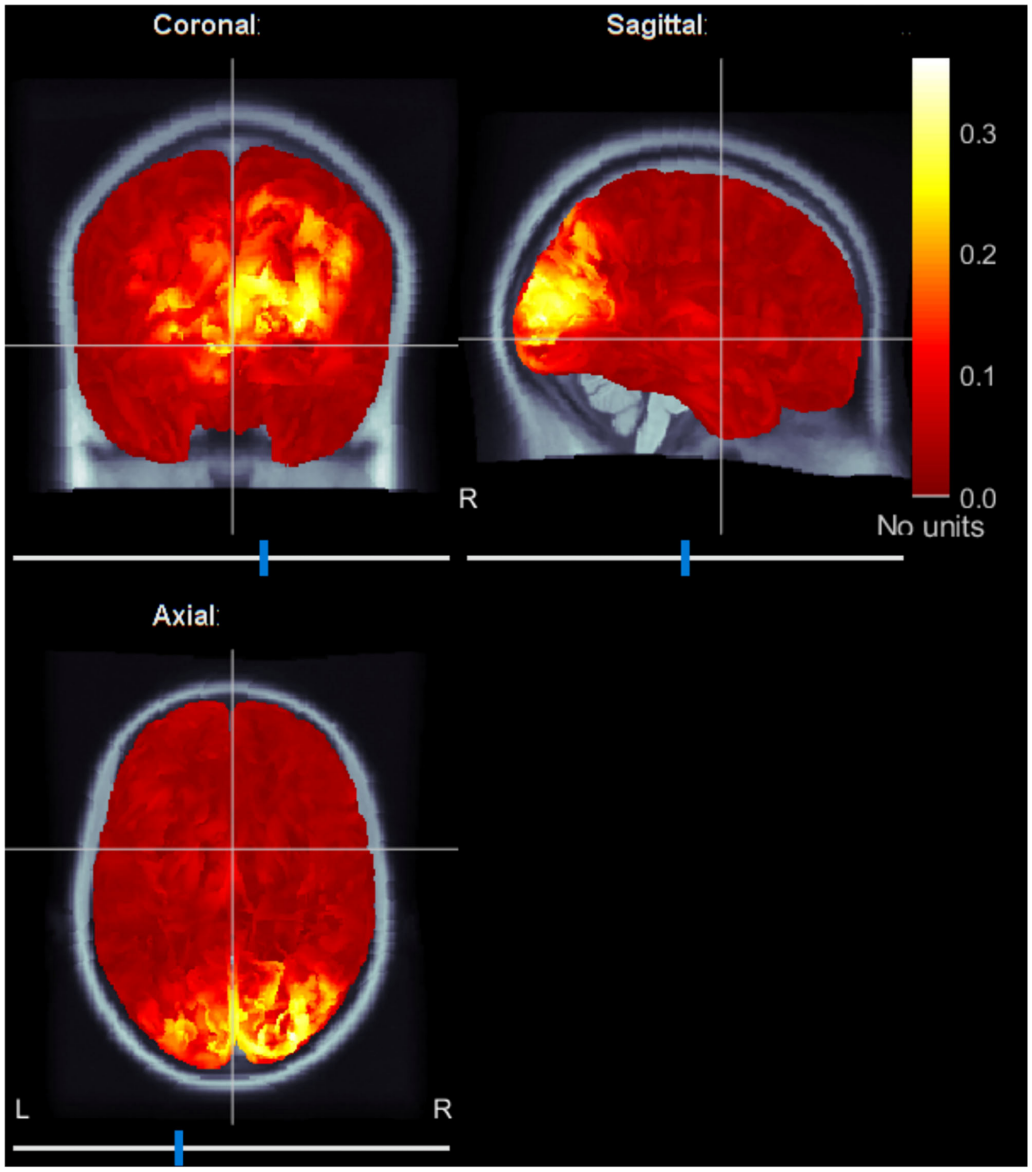
Typical source localization map using average event-related coherence. Event-related coherence for both stimulation frequencies are calculated and averaged to reveal brain sources active during the spontaneous switching between the visual perceptual states. The sources are localized in the visual cortex.

In addition, we localized brain sources averaging ERC maps at *f*_1_ and *f*_2_ frequencies. Figure 6 shows the localized brain activity in the visual cortex of one of the subjects (Subject-B). Interestingly, we observed a comparatively stronger activation in the right hemisphere, which corresponds to the left visual field. These results fall in line with the preference of the left-cube orientation.

**Figure 7:**
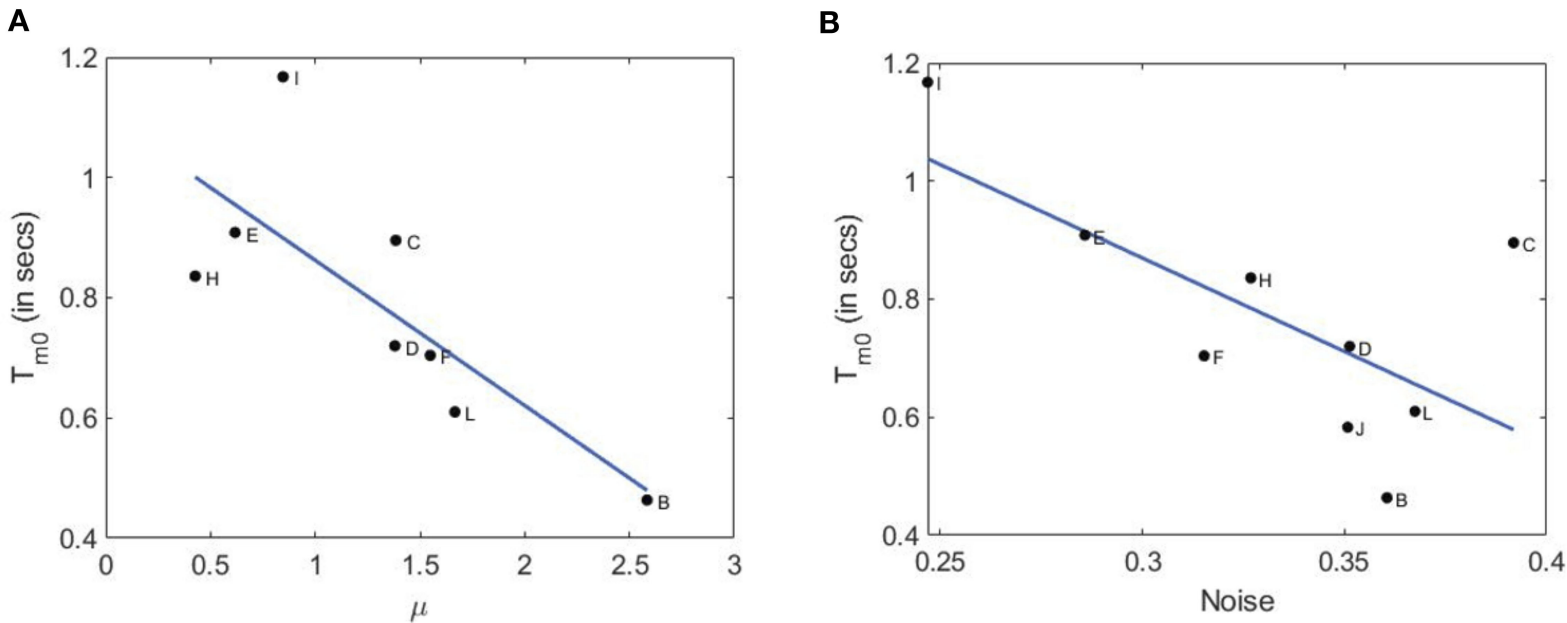
Relation of dominance time with attention performance and brain noise. **(A)** Dominance time vs. attention performance with a linear fit (root mean squared error: 0.168; F-statistics: 5.7; *p*-value: 0.0484). **(B)** Dominance time vs. brain noise with a linear fit (root mean squared error: 0.147; F-statistics: 8.95; *p*-value: 0.0242.

In the left panel of Figure 7, we plot the average modal dominance time *T*_*m*0_ = (*T*_*m*1_ + *T*_*m*2_)/2 vs. voluntary attention performance μ. As noted, only 10 out of 12 subjects participated in the second part of the experiment with an additional defaulter. Interestingly, higher attention performance leads to shorter dominance time. This is in accordance with our hypothesis that **higher attention requires a larger neuronal network to process information and make a decision**, this in turn increases neural noise since a larger number of synapses and neurons are involved (Pisarchik et al., 2019b). Finally, stronger brain noise causes more frequent switching between perceptual states or more frequent response selection and hence shorter dominance times.

To check this hypothesis, we estimated brain noise using the methodology based on phase synchronization (Boccaletti et al., 2018) as in the experiment described in our recent paper (Pisarchik et al., 2019a). In a separate set of experiments with only a single face of the cube flickering, we measured kurtosis of the probability distributions of the phase difference between the second harmonic of the flickering signal (*f*_1_) and VIF in the occipital cortex. In the right panel of Figure 7, we plot the average modal dominance time vs. brain noise (in units of inverse kurtosis). Not only do the two curves follow a similar downward trend, but subjects with higher voluntary attention also have higher noise roughly. It is important to note that brain noise was measured in a different way than in the experiment described in this paper. Nonetheless, a subject with higher (Subject-B) or lower voluntary attention capabilities (Subject-I) can be assumed to have paid a similar level of attention during the subsequent brain noise measurement experiment. As expected, these values anticorrelate, which confirms our hypothesis that **higher attention performance is associated with stronger brain noise** because a larger neural network is involved in information processing. This result is consistent with the Bialek and DeWeese theory (Bialek and DeWeese, 1995), who predicted that “the brain always finds the statistically optimal interpretation of the incoming sense data.”

## 4. Conclusion

In this paper, we have proposed novel approaches for estimating attention performance and classification of bistable perception states, based on wavelet transformation of neurophysiological brain activity. This allowed us to assess subjects in their ability to voluntarily attend to a given object and ignore the competing distractions. Owing to its non-invasive nature and relatively short conduction time, it can be used as a screening test for attentive subjects, much like IQ tests, but with much shorter conduction times.

With regard to possible applications, the developed algorithm for bistable state classification can be useful for designing new non-invasive real-time brain-computer interfaces, due to its fast computation and relative simplicity in comparison to the very heavy machine learning classification methods that require humongous computational times and larger data.

This perspective research direction requires further development. One of the possible improvements would be the combination of different methods for studying visual attention, e.g., visual-evoked spread spectrum analysis (Lalor et al., 2007) or blind source separation techniques (Tang et al., 2002). In particular, the latter method is a modification of the independent component analysis allowing to collect MEG data during cognitive tasks. Since this method does require good head stabilization, combining second-order blind identification with SSVEP would be straightforward. Filtering out the driving frequencies might allow segregation of the signal coming from different parts of the brain.

## Data Availability Statement

The raw data supporting the conclusions of this article will be made available by the authors, without undue reservation.

## Ethics Statement

The studies involving human participants were reviewed and approved by Ethics Committee of the Technical University of Madrid. The patients/participants provided their written informed consent to participate in this study.

## Author Contributions

All authors listed have made a substantial, direct and intellectual contribution to the work, and approved it for publication.

## Conflict of Interest

The authors declare that the research was conducted in the absence of any commercial or financial relationships that could be construed as a potential conflict of interest.

## Footnotes

^1^Subject-G and Subject-A did not participate in the involuntary attention experiment due to personal reasons. Additionally, there was a defaulter in each involuntary (Subject-K) and voluntary (Subject-J) attention experiments.
